# Functional connectivity in ruminants: A generalized state-dependent modelling approach

**DOI:** 10.1371/journal.pone.0199671

**Published:** 2018-06-26

**Authors:** Darcy R. Visscher, Evelyn H. Merrill

**Affiliations:** 1 Department of Biology, The King’s University, Edmonton, AB, Canada; 2 Department of Biological Sciences, University of Alberta, Edmonton, AB, Canada; University of Sydney, AUSTRALIA

## Abstract

Animal behaviour is increasingly seen as an important component in maintaining functional connectivity between patches in fragmented landscapes. However, models that explicitly incorporate behavioural trade-offs are rarely applied to landscape planning problems like connectivity. The aim of this study was to explore how state-dependent behaviour influenced functional connectivity between patches from a theoretical perspective. We investigated how inter-patch distances influenced functional connectivity using a dynamic state variable model framework. The decision making process of an individual ruminant facing fitness trade-offs in staying in its patch of origin or moving to another patch at various distances were explicitly modelled. We incorporated energetic costs and predation costs of feeding, ruminating, and resting while in the patch and for transit between patches based on inter-patch distance. Functional connectivity was maintained with isolated patches when they offered high intake and the inactivity of rumination associated with rapid gut fill resulted in reduced predation risk. Nevertheless, individuals in high energetic state often would forgo moving to another patch, whereas individuals in poor energetic states were forced to accept the cost of movement to best meet their requirements in the distant patch. The inclusion of state-dependent behavioural models provides important insights into functional connectivity in fragmented landscapes and helps integrate animal behaviour into landscape planning. We discuss the consequences of our findings for landscape planning to show how the approach provides a heuristic tool to assess alternative scenarios for restoring landscape functional connectivity.

## Introduction

Animal behaviour is regarded as an increasingly important process in conservation ecology, particularly the interaction between movement behaviour and landscape pattern [1–5]. Quantification of the structural connection of patches is no longer considered adequate for assessing the connectivity among patches. Indeed, Belisle (2005) advocated for an assessment of functional connectivity that connects a behavioural understanding of why animals are motivated to move to a new patch along with the degree to which landscapes both facilitate or impede animal movement among resource patches [6–8]. By differentially distributing individuals on the landscape functional connectivity directly influences population growth and trophic dynamics [9–13], and is critical for conservation and management of animal populations [14–19]. The motivation to move among patches comes in the form of trade-offs between rewards, such as gaining access to forage resources or mates and avoiding the risks of predation or the uncertainty in future reward. Thus, the task facing managers incorporating knowledge of animal behaviour into landscape planning is to identify and understand how factors influence whether animals stay or move among habitat patches often embedded in a “inhospitable” landscape matrix.

Although there is evidence that matrix conditions influence functional connectivity between patches [20–24], most field studies are not able to separate the influence of matrix conditions from the motivation to move among patches. Gap-crossing and homing studies involving translocated individuals often are used to measure functional connectivity, but motivation among individuals within and across studies can vary [8, 20, 25, 26]. For example, the fitness consequences of not returning to ones territory, as occurs in experiments where individuals are captured and released some distance from their territory [25], may be large. Territories and home ranges represent a large investment in time and energy in gaining local knowledge of resources, likely motivating individuals to return to them over long distances [20]. Conversely, recent work has suggested that results of translocation studies may adequately reflect routine movements within the home range [27], however, this may depend on the condition or state of the individual [28]. In contrast, quantifying trade-offs in movement among patches in heterogeneous landscapes with variable predation risk and food resources has not been well studied in at large spatial scales [22, 29–32].

Optimal foraging models (OFM) often have been used to address the trade-offs in spending time in discrete patches within heterogeneous landscapes [33–35]. Recent advances in OFM include incorporating state-based decision making, predation risk, and errors in the decision making process of the forager [36–41]. Despite these improvements, the use of OFM continues to focus primarily on within patch processes even though the risk of mortality and energetic costs of moving between patches have been shown to influence animal decisions and patch choice [26, 42–46]. Therefore, incorporating costs associated with moving through the inter-patch matrix to another patch could improve our understanding of the functional connectivity in heterogeneous landscapes [22, 47–49].

Belisle (2005) highlighted two hurdles that potentially impede the efforts of ecologists to assess functional connectivity based on animal behaviour: (1) inter-individual variation in the motivation to leave a patch, and (2) variation in the connectivity between patches as a result of anisotropic differences between patches. Despite being widely applied to a variety of ecological questions (see [50]), state-dependent behavioural models have not been used to assess functional connectivity, even though their potential usefulness seems clear [51]. State-based approaches can specifically incorporate an individual’s motivation to leave a patch based on its current state and future fitness and the trade-offs in future fitness for moving to another patch, given the costs and risks of moving. Whereas state-based models can not replace the need for empirical measures of connectivity [2, 4, 5, 52], they will provide insight into the range of possible responses that may be observed in nature to help in landscape planning.

We use a dynamic state variable model [50, 53] to assess the functional connectivity between pairs of patches of variable feeding rewards where animals are subject to energetic costs and predation risk while moving between patches and when feeding in a patch, but minimize energy costs and predation risk when inactive or ruminating in a patch. We chose to model the behaviour and patch choice of a ruminant because studies have shown that behavioural multi-tasking and digestive constraints common in ruminants may have a profound effect on their decisions [54–56]. We consider the connectivity only between pairs of patches separated in space to avoid the confounding nature of landscape configuration [57, 58], but extend these results to complex landscapes with more patches elsewhere [59]. For this assessment, we created a gradient in patch conditions that motivated individuals to move from a patch because of a positive difference in foraging rewards or a negative difference in predation risk between patches; however, whether or not they moved depended on their energetic state, energetic costs and predation risk of transit. We assumed that both the energetic cost of transit between the patches and the exposure to predation risk was positively related to distance [22, 60, 61]. Connectivity between patches was indexed as the proportion of time spent by individuals in the distant patch. Finally, we illustrate a modelling approach can be used to assess the cost of isolation of a patch from a functional perspective based on animal behaviour (i.e., the energetic equivalence of isolation), not unlike giving-up density experiments that measure the energetic equivalence of risk [62] as a heuristic tool to assess alternative scenarios for restoring landscape connectivity.

## Materials and methods

We developed a dynamic state variable model to solve the optimal policy for a generalized ruminant herbivore moving between two patches embedded within a matrix of variable predation risk [50, 53, 59]. Within each patch individuals display three possible behaviours: foraging, ruminating, and resting, which have unique consequences for their energetic reserves and gut fill, both measured in state units [54]. Whether a ruminant made the decision to move from its patch of origin (hereafter patch 1) to the distant patch (hereafter patch 2) depended on the internal state of the ruminant, the difference in the patch-specific intake, rumination, and predation rates, and the energetic costs and predation risk associated with transit between patches. The optimal policy was calculated for each scenario and at each inter-patch distance and the resultant decision matrix was used for the Monte Carlo simulations [53] where we operationally defined and measured functional connectivity as the proportion of time steps spent in the distant patch. We develop this simple model as a heuristic tool [54], not limited in scope to a particular ruminant through our use of state units, which generalized and scalable [50, 53, 54]. Our goal is to balance the value of the model for application to normative scenarios of conservation planning [63, 64] while avoiding the “curse of dimensionality” resultant from the state-by-behaviour-by-patches interaction over which the optimal solution must be solved. In what follows below, we briefly describe the relationship between a forager’s behavioural decisions and the subsequent changes to individual states, while the mathematical relationships between the behavioural decisions and subsequent changes to individual states are given in Eq 2. The parameters and variables referred to in the text are further defined and described with units in Table 1.

**Table 1:** List of model parameters including a description and the baseline conditions used for simulation purposes in the dynamic state variable model, where SU are state units.

Parameter	Description	Units	Baseline value
*m*	metabolic cost	SU/time	2
*T*	total number of time steps	time units	100
*u*	predation rate in transit	risk/distance units	0.001
*c*	energetic cost of transit	SU/distance units	1
λ	probability of finding forage	-	0.95
Variable	Description	Units	Baseline value
*α*	conversion of gut contents to energy reserves	SU/time	5
*β*	intake rate	SU/time	5
*p*	patch-specific probability of predation	risk/time	0.005
*d*	distance between patches	distance units	-

### Behavioural model

#### Ruminant behaviours and state variables

Our simulated individual ruminant forager had three discreet state-related traits (*g*; gut fill, *e*; energy reserves and *i*; location) that were updated based on the consequences of three behaviours (*b*). First, the individual could forage (*b* = 1), increasing its gut fill by a certain amount measured in state units (SU), given as a patch specific intake rate (*β*_*i*_) with a probability (λ), representing the probability of finding food. The forager also ran the risk (1 − λ) of finding no food, resulting in no addition to its gut fill. When foraging the individual’s energy reserves were reduced by a metabolic cost associated with foraging (*m*) regardless if food was found. Second, the individual could choose to ruminate (*b* = 2), which results in a conversion of gut contents into energy reserves at a patch specific rumination rate (*α*_*i*_). While background conversion may occur during both foraging and rumination, we assume for simplicities sake that these values cancel out and instead model the additional or net conversion attributable to the activity of rumination [54]. Thirdly, the individual could refrain from either of these two activities and rest (*b* = 3). Resting delayed the need to return to foraging, and the potential predation risk associated with foraging that is brought on by gut emptying through rumination. Both inactive behaviours (rumination and resting) incurred a metabolic cost equal to half the cost associated with active foraging [65]. Metabolic costs (*m*) were assumed to be behaviour specific and did not differ between the patches. We assumed that behaviours are mutually exclusive [66] and that there is no cost for switching behaviours within a patch. The simulated individual was able to pursue any of these three behaviours in one of the two patches, which were indexed as *i* = 1, 2. Besides patches differing in the intake (*β*_*i*_) and rumination (*α*_*i*_) rates they offered the ruminant, they also differ in the predation risk (*p*_*i*_) to which the individual was exposed. Gut fill (*g*) and energy reserves (*e*) were constrained between 0, which resulted in death, and a maximum value (*g*_*max*_ and *e*_*max*_) set high enough so as not to influence the behaviour of the individual close to the maximum value or serve as a constraint (100 state units, SU, for both *g* and *e*) [53]. We justify the lower constraint on gut fill due to evidence that an empty gut is known to impede further digestive function in ruminants [67].

#### Patch structure and transit costs

**Fig 1:**
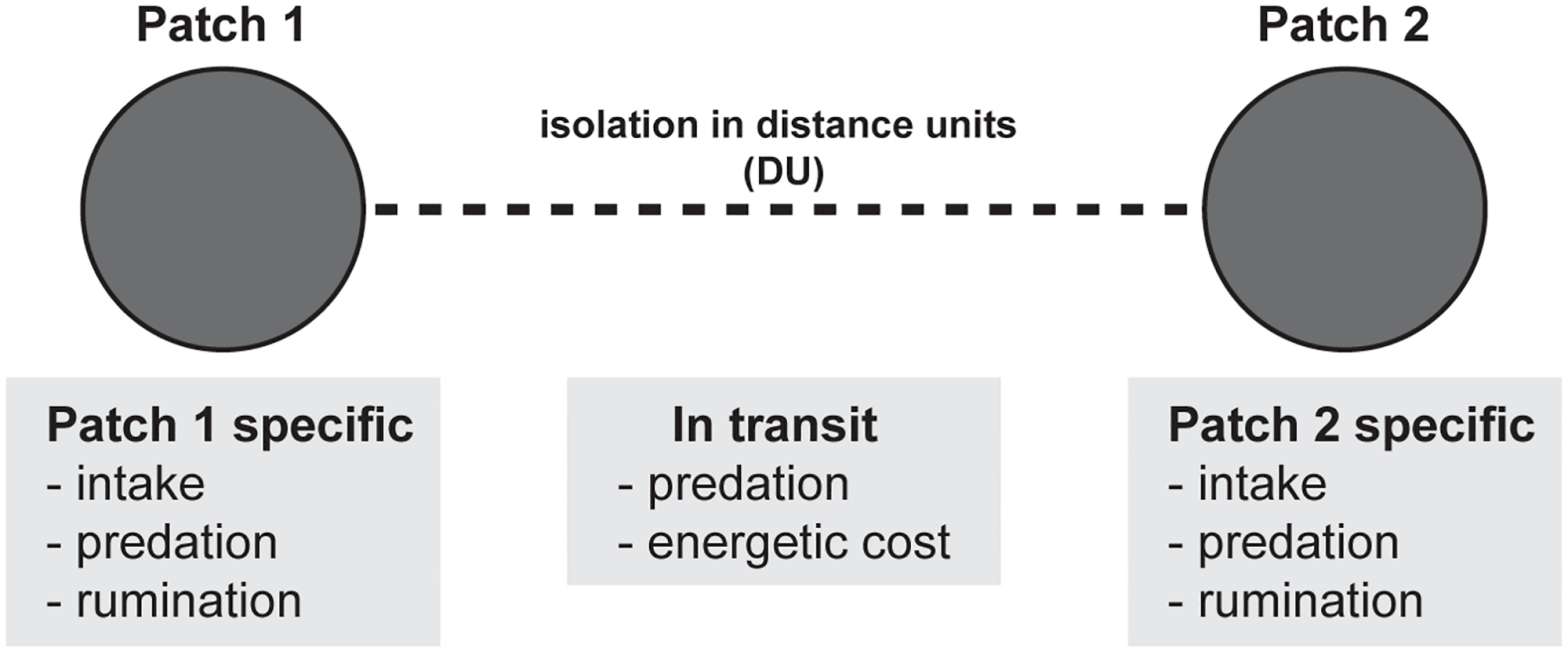
Landscape schematic. Patch-specific rates of intake, rumination, and predation risk for the patch of origin (patch 1) and the distant patch (patch 2) and the transit costs between patches as a function of the distance units (DU) between patches.

All simulated individuals started in patch 1 (Fig 1) and we imposed a motivation to move from patch 1 to patch 2 by independently varying the foraging rewards and predation risk in patch 2 relative to patch 1. Cost of transit (*c*) between patches was related to the distance between patches (*d*) such that the energetic cost for a simulated individual increased linearly with distance (*c* = 1*d), as did their exposure to predation during transit (*u* = *d*/1000; Table 1). In each simulation we used baseline values for patch 1 and varied the values of patch predation risk, intake rate, and rumination rate in patch 2, for 5 scenarios (Table 2) while incrementally increasing inter-patch distances, which are displayed in distance units (DU) representing the distance weighted energetic cost of movement between patches. If the state-dependent reward of moving to patch 2 was sufficient to overcome the transit costs (energetic cost and predation risk) then the individual would subsequently be found in patch 2. Once an individual moved to patch 2 there was no motivation to move back to patch 1 because conditions in the patches remained constant for the simulation and the relative trade-offs did not change (i.e., the two patches formed a gradient). For simplicity, movement between patches was instantaneous; as such, costs of transit between patches influenced the decision to move to a new patch but not the time spent in the two patches. The baseline conditions are given in Table 1.

**Table 2:** Simulation scenarios for functional connectivity. Patch based motivation investigates the influence of patch enhancement of the distant patch (patch 2) relative to the starting patch (patch 1), this was done by increasing the intake rate and rumination rate, and decreasing the patch specific predation rate, respectively. The remaining simulation scenarios reflected situations where movement behaviour enhanced functional connectivity over and above patch enhancement. We consider movement and anti-predator behaviour are mutually exclusive to one another. In this situation a reduction either the travel cost or the in-transit predation risk results in an increased cost or risk in the other rate, respectively. *d* is the distance from patch 1 to patch 2. In all cases patch 1 remains at baseline conditions.

Motivation	Scenario	Intake	Rumination	Patch survival	Travel cost	Travel predation
Baseline	-	5	5	0.995	0	0
Patch based	Higher intake	10	5	0.995	d	d/1000
Patch based	Higher rumination	5	10	0.995	d	d/1000
Patch based	Lower predation	5	5	0.998	d	d/1000
In-transit	Cost reducer	10	5	0.995	d/2	2(d/1000)
In-transit	Risk reducer	10	5	0.995	2d	(d/1000)/2

#### Fitness functions

Stochastic dynamic programming provides the solution to an optimal decision of patch use at each time step, which maximizes fitness over the full time period based on states given trade-offs between within-patch behaviours and transit costs between patches. Following the notation of Clark & Mangel (2000) fitness is:

$$\begin{array}{c} F\left(i,g,e,t\right)=max\left[V_{i,b}\right] \end{array}$$ (1)

where where *V*_*i*,*b*_ refers to the fitness accrued in the *i*^*th*^ patch (*i* = 1 or 2) by selecting the *b*^*th*^ behaviour (1; foraging, 2; ruminating or 3; resting) and is given for each patch and behaviour, representing their state dynamics as:

$$\begin{array}{c} \begin{array}{c} V_{1,1}=\begin{array}{c} \left(1-p_{1}\right)\left(\lambda \right)F\left(1,g+\beta_{1},e-m,t+1\right)+\\ \left(1-p_{1}\right)\left(1-\lambda \right)F\left(1,g,e-m,t+1\right) \end{array}\\ V_{1,2}=F\left(1,g-\alpha_{1},e+\alpha_{1}-\frac{m}{2},t+1\right)\\ V_{1,3}=F\left(1,g,e-\frac{m}{2},t+1\right)\\ V_{2,1}=\left(1-ud\right)[\begin{array}{c} \left(1-p_{2}\right)\left(\lambda \right)F\left(2,g+\beta_{2},e-m-cd,t+1\right)\;+\\ \left(1-p_{2}\right)\left(1-\lambda \right)F\left(2,g,e-m-cd,t+1\right) \end{array}]\\ V_{2,2}=\left(1-ud\right)F\left(2,g-\alpha_{2},e+\alpha_{2}-\frac{m}{2}-cd,t+1\right)\\ V_{2,3}=\left(1-ud\right)F\left(2,g,e-\frac{m}{2}-cd,t+1\right) \end{array} \end{array}$$ (2)

where *p*_*i*_ is the probability of a predation event (and therefore survival is 1 − *p*_*i*_) occurring in patch *i*. Additionally, we allowed the individual to assess the probability of a predation event during transit (*u*) when making the decision to travel from patch 1 to patch 2, which increased with inter-patch distance *d*. Likewise energy reserves were decreased by a cost (*c*) incurred when traveling over the inter-patch distance upon reaching the new patch. The energetic cost, in state units, of inter-patch movement for a simulated individual increased linearly with distance (*c* = 1*d), as did their exposure to a predation event during transit (*u* = *d*/1000). Thus, although costs and risk between patches depends on distance, for simplicity transit between patches occurred instantaneously and the transit costs were incorporated by discounting the potential gains of using the new patch. While instantaneous movement is unlikely in natural situations, our results reflect situations when proportionately less time to be spent moving between patches compared to time spent within patches.

Thus the fitness function at any point in time is calculated as:

$$\begin{array}{c} F\left(i,g,e,t\right)=\{\begin{array}{cc} F(i,g,e,t) & if\;t<T\\ \Phi (e,T) & if\;t=T \end{array} \end{array}$$ (3)

As the shape of the fitness function plays a crucial role in mediating the non-consumptive effects of predation [68] and to ensure that the form of the fitness function did not affect the outcome of the model, as suggested by Burrows et al. (2000), terminal fitness, Φ(*e*, *T*), was calculated two different ways [69]. In the first situation, terminal fitness as a product of increasing energy reserves was calculated as:

$$\begin{array}{c} \Phi \left(e,T\right)=e_{T} \end{array}$$ (4)

or defined as a sigmoid fitness function as:

$$\begin{array}{c} \Phi \left(e,T\right)=\frac{exp(-rep+e_{T})}{1+exp(-rep+e_{T})} \end{array}$$ (5)

where *rep* is some reproductive threshold that must be met by the energy reserves in order to increment fitness. The shape of the fitness function describes the utility of additional energy stores for the individual, given its current energetic state, and as such represents the prioritization of the fitness benefits of additional energy and remaining safe from predation. Fitness as a linear function of energy reserves tends to prioritize gaining additional energy reserves over avoiding predation. Conversely, fitness as a sigmoid function prioritizes remaining safe over gaining additional energy reserves, when above some reproductive threshold *rep*. These two fitness functions generally represent the distinction between a rate maximizing and time minimizing foraging strategy [70, 71], which in ruminants, may be related to season or sex differences and shifting energetic requirements and life history traits [72–76]. Notice that in both cases, when *t* = *T* fitness is dependent only on energy reserves, not gut fill. We assume that any remaining forage in the gut at the end of the time horizon is no longer useful for an individual. A full list of parameters and variables, their description and values are given in Table 1.

### Simulations and scenarios

The optimal policy calculated from the stochastic dynamic programming equation (Eq 1) through backward iteration was calculated uniquely for each scenario and at each inter-patch distance [53]. The resultant decision matrix was used for the Monte Carlo forward iteration procedure [53], simulating 100 individuals, starting at each possible discreet combination of gut fill and energy reserves (100 x 100 or 10000 unique state combinations in total) for the same time period (*T* = 100) to account for the probabilistic nature of predation. Each individual followed the optimal policy (calculated from Eq 1) and updated its state at each time step unless it died from predation or starvation. We investigated the trade-offs in foraging opportunities within patches and the costs for between patch transit with a set of five simulation scenarios. We compare these scenarios qualitatively to determine their impact on promoting functional connectivity. In all cases, patch 1 had baseline characteristics (Table 1) and only distance to and the conditions in patch 2 were altered.

For each simulation we recorded the proportion of time individuals spent in patch 2 and used the average proportion of time spent in patch 2, from the 100 simulations at each combination of states, as a metric of functional connectivity between patches. Because the initial distribution of individuals with energetic and gut-fill states was the same among simulation scenarios, a high mean proportion of time spent in patch 2 reflected high patch connectivity or little resistance to moving among patches due to low transit costs. Within the context of our simulations this metric of functional connectivity is appropriate, however, when individual states are not known this metric may not convey useful information about functional connectivity [8].

In the first set of simulations we modelled patch-based motivation (see ***Patch quality and functional connectivity*** below) to maintain functional connectivity under different fitness functions by increasing intake or rumination rate, or lowering the predation risk of patch 2 while keeping transit costs constant at baseline values (but still dependent on inter-patch distance).

Second, we modelled the effects of functional connectivity under varying energetic cost and predation risk for in-transit movement (see ***Transit costs and functional connectivity*** below). In these scenarios we kept patch 2 characteristics constant (but with high intake) and varied only transit costs. For simplicity, we assumed that the in-transit predation costs were inversely related to the in-transit energetic costs. We modelled in transit behaviour as a trade-off, individuals were able to reduce one of the transit costs (either energetic cost or predation risk) at the expense of the other. We took this approach because the probability of predation while moving between patches depends not only on the magnitude of the risk but also on the duration of risk exposure. For example, an individual may move more quickly among patches to minimize predation exposure, but it will incur higher energy costs [22, 60, 61, 77]. The patch values and transit costs for each scenario are given in Table 2 and a representation of the patches is illustrated in Fig 1.

Third, we compared functional connectivity dependent on whether inactive behaviours (i.e., resting and ruminating) provided reduced predation risk relative to exposure during active foraging (see ***Behavioural refuge and functional connectivity*** below). We compared outcomes of the scenarios where inactive behaviour were completely effective at reducing predation risk to the situation where they did not reduce predation risk relative to active foraging.

Lastly, we conducted simulations to titrate the level of energetic gain required in patch 2 to uncover the energetic equivalence of patch isolation (see ***Titrating the cost of isolation*** below). We did this by using a set of inter-patch distances over which we see no functional connectivity or use of the distant patch, and consecutively increase the energy intake rate of the distant patch until functional connectivity was re-established. This scenario builds upon the methodology established by giving up density (GUD) experiments, which titrate the additional food required to equalize animal use between safe and risky patches, applied to the problem of patch isolation [29, 62, 78, 79].

## Results

In general, the form of the fitness function influenced the degree to which functional connectivity was maintained, as well as which individuals maintained connectivity. Individuals modelled with a linear fitness function maintained, on average, and across all scenarios, functional connectivity over greater inter-patch distances than individuals modelled with a sigmoid fitness function. The state of the individuals which maintained functional connectivity differed between those modelled with a linear or sigmoid fitness function. Transit to patch 2 occurred in individuals of all initial states when modelled with a linear fitness function as it provided a quicker means of increasing the energy reserves than staying in the patch of origin. In contrast, when modelled with a sigmoid fitness function, only individuals in “poorer” state (i.e., those below the reproductive threshold, *rep*) made significant use of patch 2, individuals with high gut fill and energy reserves most often choose not to move because they were able to meet their fitness goals in patch 1.

The form of the fitness function also determined the average survival of individuals. As an example, at a distance of 2 DU between patches, individuals modelled with a linear fitness function had a lower average rate of survival (86%) than individuals modelled with sigmoid fitness functions (96%). The difference in survival was not solely due to the risk of predation associated with transit between patches but also resulted because these individuals rarely used inactivity as a within-patch behavioural refuge.

### Patch quality and functional connectivity

**Fig 2:**
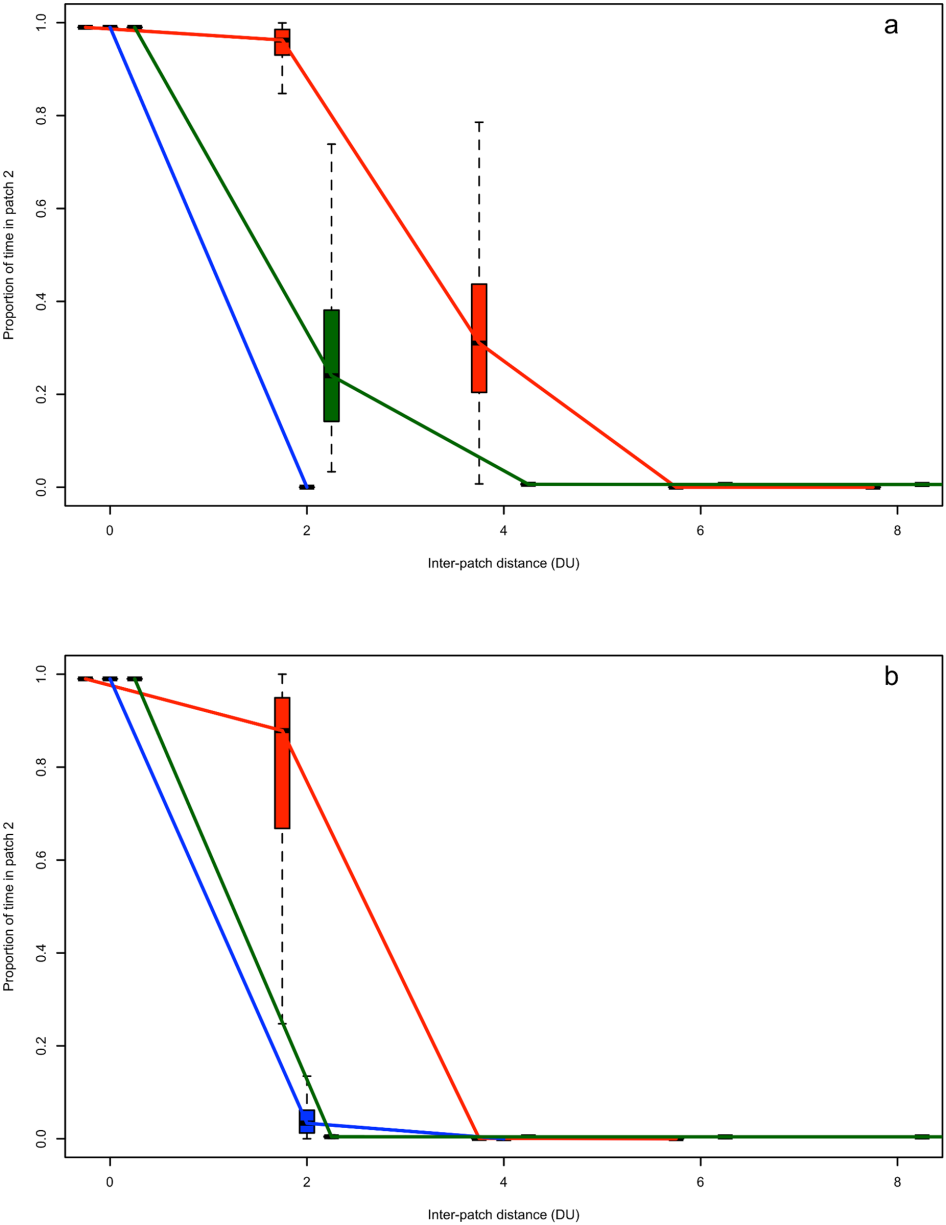
Patch based scenarios of functional connectivity. Functional connectivity measured as the proportion of time spent in the distant patch (patch 2) with respect to increasing inter-patch distances (DU) for individuals modelled using a linear (a) or sigmoid (b) fitness function. The red boxes and line indicate where motivation to move from patch 1 (origin) to patch 2 is maintained by higher intake in patch 2, the blue boxes and line refer to the motivation to move to patch 2 maintained by a reduction in predation risk, and the green boxes and line refer to the motivation to move to patch 2 maintained by a higher rumination rate. These patch based scenarios are given in Table 2, while within each scenario all other parameters held constant at baseline values (see Table 1).

Overall connectivity between patches declined with increasing distance between the patches, but the rate at which this occurred was influenced by the form of the fitness function. When a linear fitness function was used, energetic gain was prioritized above predation risk for a majority of states. Consequently, connectivity was maintained over relatively large distances compared to when fitness was modelled with a sigmoid function (Fig 2a) because individuals were motivated to move to a new patch, particularly when intake rates were high. The difference in intake rates between patches had a bigger impact on connectivity relative to an increase in rumination rate or a decrease in within-patch predation rate (Fig 2a).

In the case of individuals modelled with a sigmoid fitness function, safety was prioritized over the acquisition of energy for any individuals exceeding the reproductive threshold. As a result, individuals ceased to move to patch 2 over shorter distances relative to those modelled with a linear fitness function (Fig 2b). Instead, individuals tended to “make ends meet” in the patch of origin when the patch of origin was sufficient for maintaining their state at the baseline reproductive threshold. Raising the reproductive threshold increased the motivation to move, resulting in more individuals moving to patch 2 when it contained increased foraging opportunities. Likewise, reducing the baseline conditions in patch 1 such that the reproductive threshold could not be maintained (e.g., intake was below metabolic costs) resulted in more movement to patch 2 when patch 2 provided intake rates that exceeded metabolic costs. Because fitness was dependent on the acquisition of energy connectivity was maintained over the greatest inter-patch distances when intake rates were increased in patch 2 (Fig 2b), a decrease in patch-specific predation or an increase in rumination rate had very little effect on connectivity (Fig 2b).

### Transit costs and functional connectivity

**Fig 3:**
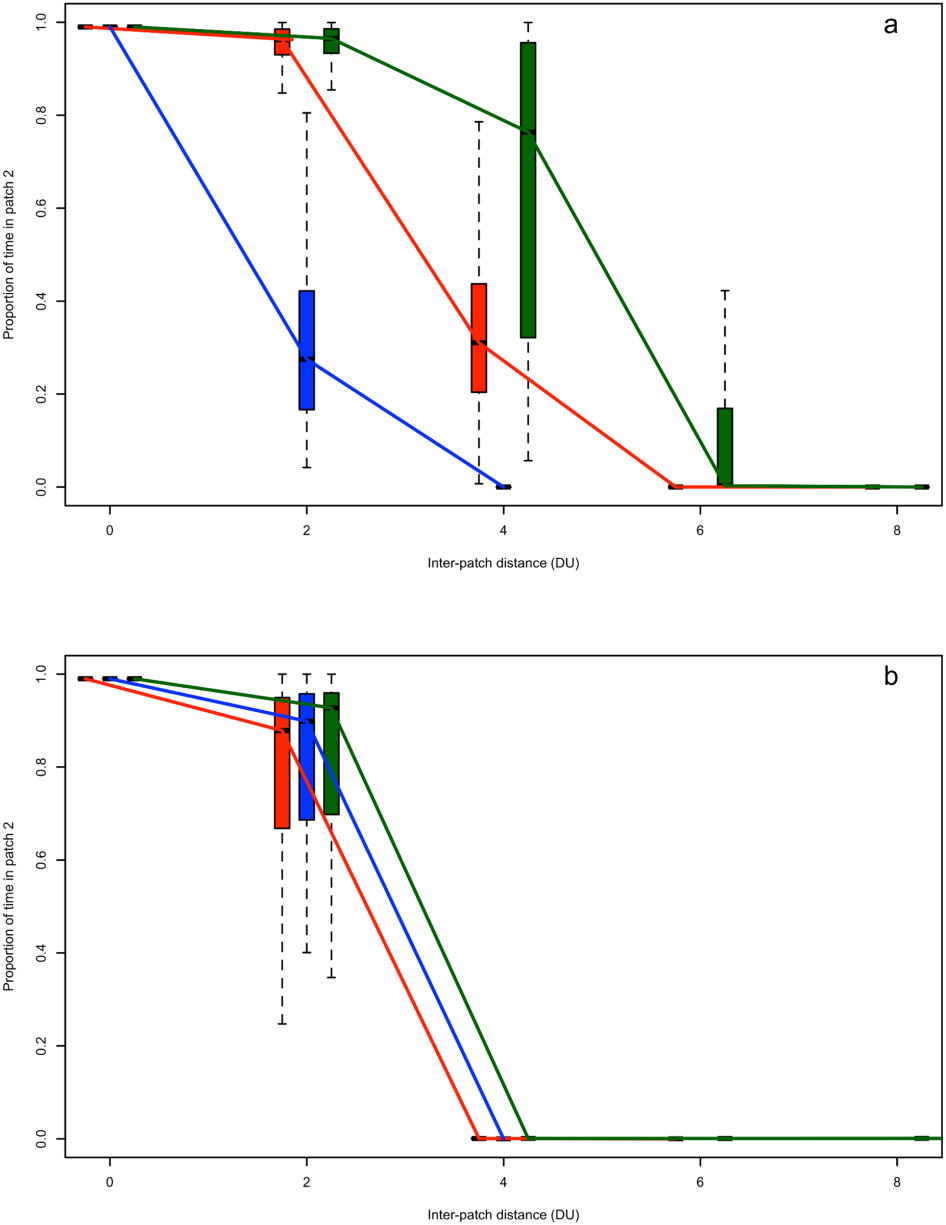
Transit based scenarios of functional connectivity. Effects of transit costs on functional connectivity for individuals using a linear (a) or sigmoid (b) fitness function given a 2x higher intake rate in patch 2 than in patch 1 when individuals can alter their in-transit behaviour. The red boxes and line indicate the connectivity under baseline energetic cost and risk of predation for transit (Table 1), the blue boxes and line refer to the connectivity under 2x baseline energetic cost and 1/2 risk of predation for transit, and the green boxes and line refer to 1/2 energetic cost and 2x predation risk for transit. These in-transit scenarios are given in Table 2, while within each scenario all other parameters held constant at baseline values (see Table 1).

For individuals modelled with a linear fitness function, behaviours that resulted in a decreased transit cost facilitated connectivity over larger inter-patch distance over-and-above changes in the quality of patch 2 (Fig 3a). Individuals who reduced the transit predation risk at the expense of increased energetic cost reduced the connectivity relative to changes in the quality of patch 2 (Fig 3a). In contrast, for individuals modelled with a sigmoid fitness function, when either the energetic cost or predation risk during transit was reduced, at the expense of the other transit rate, there was little change in connectivity over-and-above that produced by the baseline patch conditions (Fig 3b). However, under both fitness functions, when the costs associated with transit were increased further and independently, functional connectivity depended on the specific trade-off between energetic cost and the predation risk of transit. That is, individuals that matched their in-transit behaviour to the greatest cost (energetic or predation) maintained the highest levels of functional connectivity.

### Behavioural refuge and functional connectivity

**Fig 4:**
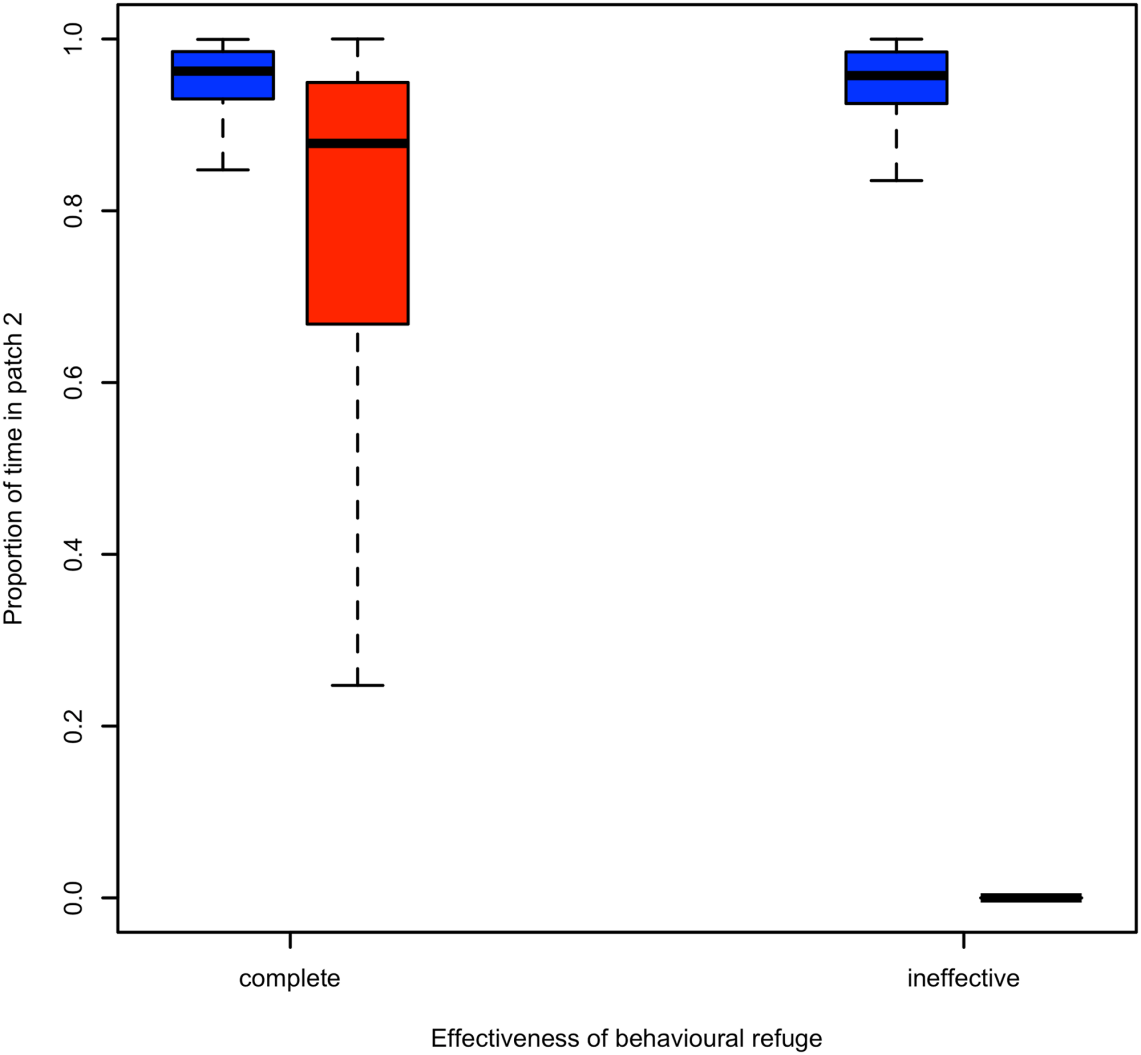
Functional connectivity and the effectiveness of the behavioural refuge. The influence of the behavioural refuge (effectiveness of anti-predator behaviour) during inactivity on functional connectivity, measured as the proportion of use of patch 2, when inactivity forms a complete refuge (i.e., predation rate during inactivity = 0) or inactivity conveys no anti-predator benefit (i.e., predation during inactivity = predation risk during foraging). Individuals modelled with a linear fitness function are given in the blue boxes, while individuals modelled with a sigmoid fitness function are given in the red boxes. Inter-patch distance is 2 DU and patch 2 has an increased intake rate relative to patch 1 while all other values remain constant at baseline values.

The extent to which safety during inactive behaviours (rumination and resting) influenced connectivity was assessed for the situation only where movement to patch 2 was motivated by increased intake. For simplification, we compared outcomes only among individuals modelled with a linear and sigmoid fitness function at one patch distance (i.e., 2 DU). We found that the effectiveness of within patch anti-predator behaviours (i.e., inactivity) had little to no effect on the functional connectivity of patches for individuals modelled with a linear fitness function. For individuals modelled with a sigmoid fitness function there was a large difference in connectivity (Fig 4). When individuals could use inactivity to form a complete behavioural refuge (i.e., through effective anti-predator behaviours) high levels of patch connectivity were maintained. When inactivity was ineffective and individuals were exposed to the same patch-specific risk of predation, as when actively foraging, connectivity ceased completely as both patches were equally risky and the additional movement cost was not warranted, rather individuals made “ends meet” in the first patch.

### Titrating the cost of isolation

**Fig 5:**
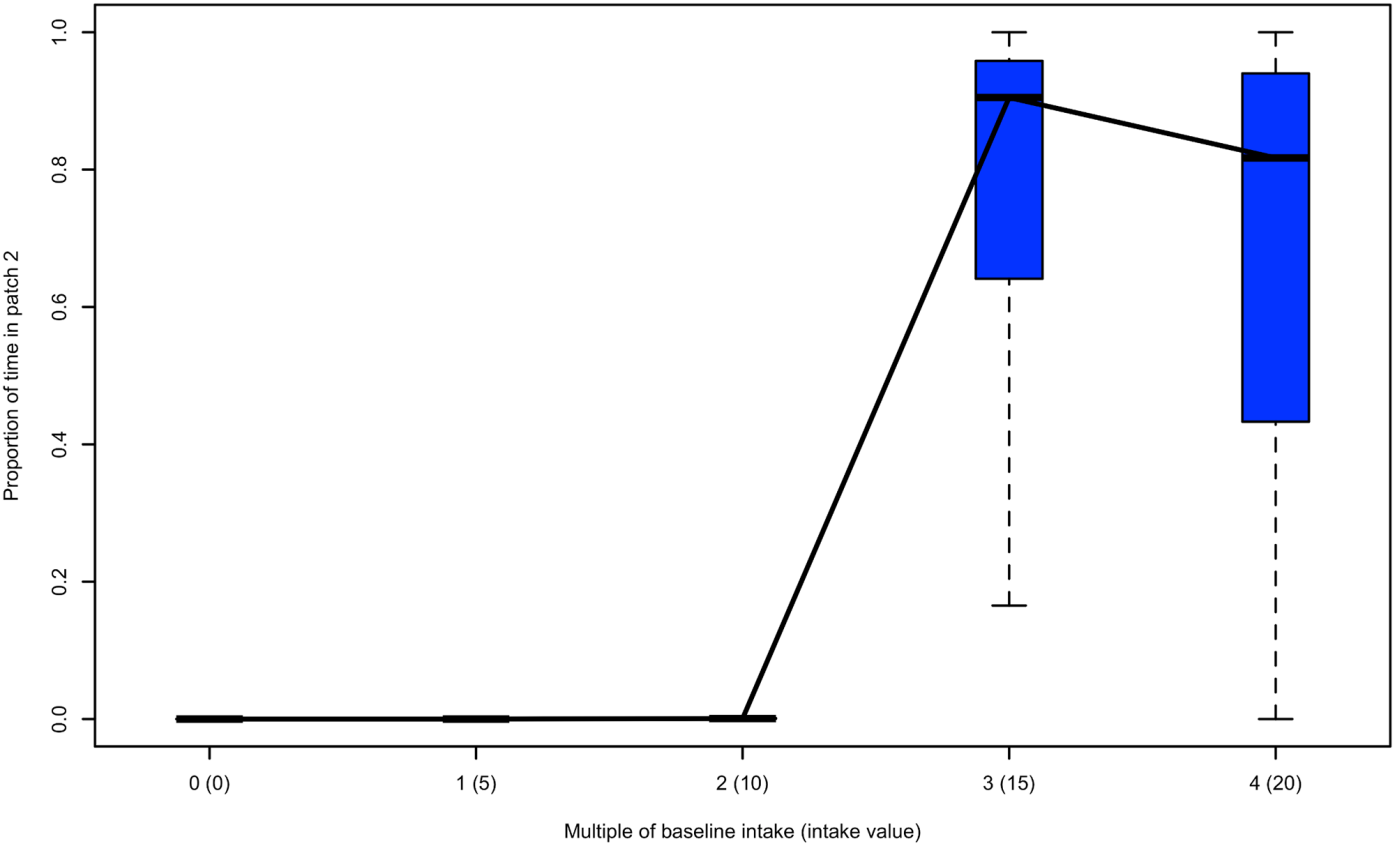
Titrating the energetic equivalence of patch isolation. Example of the energetic equivalence of isolation as assessed by a titration experiment. This represents the marginal rate of substitution of energy for isolation and indicates the incentives needed in the second patch to restore functional connectivity. At an inter-patch distance of 4 DU, increasing motivation was presented to individuals (simulated with a sigmoid fitness function) in the form of increased intake, given as multiples of baseline values (and absolute intake values). The proportion of time spent in the isolated patch (patch 2) was recorded. There is non-zero use of the patch at an intake of 10 and substantial use at an intake of 15.

Using our approach, we assessed how willing a ruminant was to accept the costs of transit by determining the rewards necessary to increase use of the distant patch. This allowed us to determine what factors may most readily restore functional connectivity in already fragmented landscapes. We initially assumed that animals prioritized safety (i.e., sigmoid fitness function) and exposed them to a range of patch conditions that represented when individuals typically ceased to move to patch 2. (i.e., 4 DU; energetic cost of 4 and an in-transit predation risk of 0.004). We then consecutively increased the intake rate in patch 2 to determine at what intake value at least 50% of the individuals were motivated to overcome the costs of transit to move to patch 2. Under the assumption of a sigmoid fitness function, we found that intake rates in patch 2 had to be increased approximately 2.5 times before individuals spent over 50% of their time in patch 2 (Fig 5). Assuming a linear fitness function, an approximately 2-fold increase in intake in patch 2 resulted in over 50% of an individual’s time in patch 2. The difference in fitness functions was due to individuals modelled by the linear fitness function already prioritizing energetic gain by using patch 2 approximately 30% of the time under the simulated conditions.

## Discussion

Maintaining or restoring landscape connectivity is an important component of conservation [15, 19]. We demonstrated with the use of dynamic state variable models that the state of the animal, the foraging strategy (e.g., energy maximization or foraging time minimization to avoid predation as reflected in the fitness function), and the difference in patch quality all had a strong influence on the motivation to move to a new patch, which we considered reflected functional connectivity. Nevertheless, four conclusions come from our simulations. First, consistent with optimal foraging theory [35, 39, 78] where there is minimal opportunity to forage in the inter-patch matrix, animals may not be motivated to move from a low quality patch if the costs of transit are high unless improved intake rates in the distant patch offer the possibility of recouping at least the cost of movement. While our model focused on a pair of patches, the result we describe scale to multi-patch situations where patch adjacency and context play a role in distributing use across patches [59]. Enhancement of energetic gain through forage improvements, such as forage seedings, burning, additional patch creation, or selective forest cutting, is a common management strategy to encourage the use of areas for many wild ruminants [80–83].

Second, if activity during foraging increases the risk of predation, the additional foraging required to offset the energetic cost of transit may result in decreased survival. As a result, even if patches provide increased foraging opportunities, the associated high risk of predation could create an attractive sink [84, 85]. Delibes et al. (2001) and Kristan (2003) attributed movement to attractive sinks to poor perception or suboptimal decision making in patch selection. Alternatively, our results indicate an individual’s previous energetic state may determine its willingness to move among patches because it can afford short-term energetic losses (i.e., the “silver spoon” effect *sensu* [86]), which are subsequently recouped if distant patches have higher intake offering higher energetic gain [87, 88]. Our results support the accumulating evidence from field and simulation studies suggesting that individual state has a strong influence on animal decisions [1, 28, 51]. For example, Zollner & Lima (1999, 2005) showed a strong effect between the amount of energy reserves and the probability of successful dispersal, where individuals with higher reserves were able to move farther to locate a patch because they were able to sustain the cost of anti-predator defences during dispersal. However, field studies examining how animal condition influences frequency of movements among patches at either small (habitat use) or large scales (migration) remain largely unexplored [89–91].

Third, reducing the predation risk of the distant patch had the least effect on maintaining connectivity. The effect may have been due to the overall low predation risk within the landscape conditions we simulated. In situations where the differences in predation risk between patches is large, such as when some patches are refuges from predation [92, 93], the willingness to move to a safe patch may extend connectivity over a wide range of inter-patch distances. For example, one hypothesis for migratory behaviour in ruminants has been predator avoidance [94, 95]. The influence of predation risk within a patch also may depend on the ability of individuals to modify risk using anti-predator behaviours, once in a new patch. We assumed that individuals used inactivity to reduce predation risk within patches because it has been reported in field studies [96]. When inactivity, such as when ruminant lie down to ruminate, reduces predation risk, individuals using a time minimizing foraging strategy (i.e., sigmoid fitness function) to avoid predation gained little from moving to a new patch and did not expose themselves to any added costs of moving between patches. However, the effectiveness of the anti-predator behaviour depended on forage resources. The potential benefit of inactivity to reduce risk increased when intake rate was high, because it enabled an individual to reach their fitness goals in a short time reflecting a time minimizing strategy. The effectiveness of other anti-predator behaviours may also depend on forage conditions; for example, vigilance may reduce intake only when foraging is encounter-limited [97, 98], and forming large groups may be possible only where resources are high [99]. Incorporating other anti-predatory behaviours like vigilance and grouping behaviour into models for a general assessment of anti-predator behaviours on connectivity may be possible given the recent emphasis in field studies to understand the trade-offs of these behaviours [97, 100–103].

Similarly, the effectiveness of a ruminant’s anti-predator behaviour maybe further enhanced by an individual locating itself in refuge habitats infrequently used by predators. Elk are known to increase their use of foraging habitat in proximity to human activity to avoid predation by wolves [104–106]. The proximity of these individuals to humans may lead to habituation and poses clear management implications, including changes to migratory behaviour [104, 107]. Our results suggest that in these cases, individuals may be reluctant to move and would rather “make do” with the current foraging patch due to a prioritization of safety [59]. The shape of the fitness function in our simulations, in part, determines how individuals prioritize safety. Thus, managers facing problems with habituation may need to consider seasonal or sex differences in energetic requirements in determining strategies for removing individuals from proximity to humans as well as making those locations “unappealing” for individuals to return to [74–76, 104].

Fourth, functional connectivity was maintained over farther inter-patch distances when individuals were able to reduce the energetic cost of moving among patches even at the expense of increased predation risk. However, our simulations show that the degree to which connectivity was maintained also depended on the foraging strategy specified by the fitness function with the actual outcome determined by the specific conditions encountered when moving between patches. In nature, ruminants may have shifting strategies for meeting fitness goals that influence the behavioural trade-offs they make. Evidence from seasonal changes in habitat selection suggest that individuals may differentially prioritize safety and the acquisition of energy at various times of the year [76, 108]. Likewise, sex and differences in reproductive status may also effect the ability of individuals to trade-off predation risk and foraging opportunities either with anti-predator behaviours within a patch or with patch selection [74, 108–112]. Dussault et al. (2005) noted that there was variation in patch selection between individual caribou, which they attributed to the sex and reproductive status of the individual, which represented their motivations for the trade-off between predation risk and foraging opportunities. Additionally, Gustine et al. (2006) noted that the ability of individual caribou to respond to predation risk was condition-dependent: females in poor condition took higher risks in order to access forage, as they could not afford to avoid predation. These results highlight the importance of sampling trade-offs in foraging and predation risk multiple scales and the need to integrate between scales through the inclusion of behavioural motivation, which may facilitate trade-offs between predation risk and foraging opportunities [43, 113, 114]

The interaction between movement and the efficacy of anti-predator behaviours while moving between patches has not been well documented nor even explored in a modelling framework (but see [22]). Our results suggest that if individuals are able to match their transit behaviour to the specific conditions they encounter, functional connectivity can be maintained even over long distances, yet general predictions may not be possible because outcomes depend on the specific landscape situations. The same variability is evident in field observations with some field studies showing individuals increase movement rates in areas of high predation risk [77, 115], whereas others suggest that movements decrease in risky areas [61, 116, 117]. It is possible that decreased movement rates may facilitate anti-predator strategies. For example, McAdam & Kramer (1998) found that squirrels and chipmunks used intermittent pauses when moving into riskier habitats, presumably for added vigilance. If anti-predator strategies are mutually exclusive from movement then minimizing either movement costs or the in-transit risk of predation becomes a trade-off [22, 30, 61, 118]. Understanding whether animals can mitigate the cost of rapid movement with behaviours while in transit remains a fruitful area for study, and may lead to a better assessment of the role that behaviour plays in functional connectivity in complex landscapes [59, 91].

## Conclusion

We have shown how the state-dependent modelling framework can be used as a heuristic tool [54] for investigating functional connectivity by assessing the “behavioural permeability” based on movement cost and predation risk. While we have presented a simple state-dependent model in the hopes of stimulating further development and use of this framework for understanding individual motivation to maintain landscape connectivity, we are confident that our results are generalizable and scale [8, 59]. In particular, a state-dependent approach can be used to compare normative landscape scenarios that portray alternatives futures so that managers restoring landscapes may experiment with inventing landscape patterns that are expected to function according to ecological and society values [64]. We illustrate that when applied to connectivity analysis it was sensitive to changes in the relevant behaviours that may affect the functionality of moving within a heterogeneous environment. Further, by addressing the question, “What additional state-dependent motivation is required to overcome the costs associated with patch isolation?”, it may not only provide guidance on how to potentially improve restoration options for species in fragmented landscapes, it may lead to new ways to configure landscapes to improve functionality [8, 22, 47, 64, 119–121]. The prospective scenarios our approach offers may be more useful than projective scenarios, which describe the future, when uncertainty is large and uncontrollable [63], and lead managers to be more inventive than they might otherwise be in meeting ecological and societal mandates. However, like all models, dynamic state variable models require simplification of relevant biology, and it initially may be difficult to parameterize the model for a particular situation; for example, it requires more realistic landscape context than the 2-patch scenario presented here [122]. Despite this, spatially explicit, state-dependent models using dynamic programming are becoming more commonly used to assess space use of a range of species where individuals face a range of behavioural trade-offs that have fitness consequences [41, 123–126].

## Supporting information

S1 Appendix Stand alone program to solve the SDP algorithm based on user specified states and state dynamics and to do stochastic simulations based on the solved optimal policy. (EXE)

S2 Appendix Example XML configuration file used by the stand alone program to run a user specified scenario. (XML)
